# Gene therapies and COVID-19 vaccines: a necessary discussion in relation with viral vector-based approaches

**DOI:** 10.1186/s13023-021-01958-3

**Published:** 2021-07-16

**Authors:** Angel Aledo-Serrano, Antonio Gil-Nagel, Julian Isla, Ana Mingorance, Fernando Mendez-Hermida, Ruben Hernandez-Alcoceba

**Affiliations:** 1 Genetic Epilepsy Program. Neurology Department, Ruber Internacional Hospital, Madrid, Spain; 2 Dravet Syndrome European Federation, Split, Croatia; 3 Dracaena Consulting, Madrid, Spain; 4 Vaccines and Advanced Therapies, Spanish Agency of Medicines and Medical Devices, Agencia Española de Medicamentos Y Productos Sanitarios (AEMPS), Madrid, Spain; 5 Gene Therapy Program CIMA, IdiSNA, Navarra Institute for Health Research, University of Navarra, Pamplona, Spain

**Keywords:** SARS-CoV-2, Adenovirus, AAV, Genetic diseases, Rare diseases, Pandemic, Advanced therapies

## Abstract

The COVID-19 pandemic is adding an unanticipated concern for those affected by genetic diseases. Most of the new treatment achievements for these patients are made possible as a result of advances in viral-based products. Among them, adenoviruses (AdV) and especially adeno-associated viruses (AAV) are important players. The concerns and the conversation around this issue have increased as COVID-19 vaccines approach the market. What if the viral vectors become the mainstream strategy for vaccine development? Will the immune response elicited against the vector compromise the efficacy of future gene therapies? Patients with genetic diseases and patient advocacy groups are requesting information to the medical community about the potential impact of these vaccines in future gene therapy treatments, and physicians and scientists are not able to provide satisfactory answer yet. Importantly, the frequency of cross-reactivity among different AAV serotypes can be as high as 50%. This would have potential implications for patients with genetic disorders who could benefit from gene therapies, often coming in the form of AAV-based gene therapies. As in many other aspects, this pandemic is challenging our capacity to coordinate, plan ahead and align different medical objectives. In this case, having such conversation early on might allow us to make the right choices while we are still on time.

## Main text

Patients with severe genetic diseases and their families are in need of accurate medical information for the day-to-day care of their disorder, as well as for longer-term management planning. Often, this available information is insufficient for their needs and go beyond what health system can provide, which leads patients and families to become organized as patient associations, in order to gather information about treatment options and research advances. For families with children with genetic rare diseases, keeping their child as healthy as possible until a disease-modifying therapy becomes available becomes a priority. Recent successes in developing such therapies for genetic rare diseases fuel the patient community with excitement, as they continuously await for new biomedical research to produce gene therapies for their disorders. Most of the new treatment achievements are made possible as a result of advances in viral-based products. Among them, adeno-associated viruses (AAV) are currently considered crucial vectors applications, as reflected in the number of AAV medicinal products approved by the European Medicines Agency (EMA).

In this context, the COVID-19 pandemic is adding an unanticipated concern for those affected by genetic diseases: what if the viral vectors become the mainstream strategy for vaccine development? Will the immune response elicited against the vector compromise the efficacy of future gene therapies? Should new treatment guidelines be implemented in order to facilitate orphan disease affected patients to receive alternative types of vaccines? Should youngest patients also be given the opportunity to be immunized with non-vector vaccines, since they are theoretically more susceptible to present gene related diseases during their lifetime than older vaccine candidates? These are not trivial questions, since the presence of neutralizing antibodies against the virus used as a vector is an exclusion criterion for many clinical trials with viral-based gene therapies [1].

The concerns and the conversation around this issue have increased as vaccines approach the market. Patients with genetic diseases and patient advocacy groups are requesting information to the medical community about the potential impact of these vaccines in future gene therapy treatments, and physicians and scientists are not able to provide satisfactory answer yet. Firstly, we observed the development of adenovirus-based vaccines against COVID-19 (such as those from Astra-Zeneca, CanSino, Janssen and Gamaleya center). Adenoviruses and AAV vectors employed in gene therapy do not share relevant structural proteins, excluding the possibility of cross-reactivity among them. However, it should be noted that adenoviruses are a relevant tool for the treatment of diseases where a larger DNA construct is required and AAV do not have the required cloning capacity [2]. Since these High-Capacity adenoviral vectors (HC-AdV) have not entered clinical trials for genetic diseases yet, AdV types currently used as COVID-19 vaccines can be avoided in the final product, following relatively straightforward methods [3].

Nevertheless, recent news about AAV-based treatments and vaccines being considered for COVID-19 have reignited the concerns and needs for information from the rare disease patient community. In one approach, AAVs can be used to transfer the genetic information needed to produce neutralizing antibodies against SARS-CoV-2. This strategy could obtain a fast protection against the virus, even in subjects with weakened immune system, since the antibodies would be directly produced by cells in the respiratory tract, not the lymphocytes [4]. In another approach, AAVs could be designed to express critical domains of the SARS-CoV-2 Spike protein [5], following the strategy of the first generation of adenovirus-based vaccines. In this case, the high thermal stability of AAV particles facilitates the logistics of global vaccination campaigns, by alleviating the need of a highly demanding cold chain in areas with weak civil infrastructures. But this undeniable success against COVID-19 would not come alone. The global vaccination would create a parallel immunization against the chosen AAV serotype. Importantly, the frequency of cross-reactivity among different AAV serotypes can be as high as 50% [6, 7]. This would have potential implications for patients with genetic disorders who could benefit from gene therapies, often coming in the form of AAV-based vectors. The good news is that the repertoire of AAVs is remarkably wide, and careful selection can identify candidates with optimal properties as vaccines and low risk of cross-reactivity with commonly used gene therapy vectors [5].

In conclusion, the potential interference between gene therapy and virus-based vaccines deserves a careful consideration and discussion involving the patient community. This is a adequate moment to do so, since current vaccine candidates are not expected to cause such interference. Should people with monogenic diseases preferentially receive, or be given the option to be immunized COVID-19 vaccines based on non-viral platforms, such as the mRNA vaccines? A careful consideration of the impact on the use of vectors for vaccine production and the plausible negative impact in the development of suitable gene therapy medicinal products, should be on top of the scientific and regulatory table discussion in order to facilitate access to patients suffering monogenic diseases and also prevent unnecessary delays in future vector vaccines development. Hopefully, this issue will become less relevant when new generations of non-viral vectors will start to show efficacy in the treatment of monogenic diseases [8]. As in many other aspects, this pandemic is challenging our capacity to coordinate, plan ahead and align different medical objectives. If patients affected with rare diseases are to be granted the possibility of choosing non-viral vectored vaccines, additional discussion is also needed with regards on how this information should be made publicly available, since it cannot be ruled out that additional irrational and non-scientific based opinions could spread among the public against safety of viral vector vaccines. In this case, having such conversation early on might allow us to make the right choices while we are still on time.

## Data Availability

Not applicable.
